# A principal components analysis of factors associated with successful implementation of an LVAD decision support tool

**DOI:** 10.1186/s12911-021-01468-z

**Published:** 2021-03-20

**Authors:** Kristin M. Kostick, Meredith Trejo, Arvind Bhimaraj, Andrew Civitello, Jonathan Grinstein, Douglas Horstmanshof, Ulrich P. Jorde, Matthias Loebe, Mandeep R. Mehra, Nasir Z. Sulemanjee, Vinay Thohan, Barry H. Trachtenberg, Nir Uriel, Robert J. Volk, Jerry D. Estep, J. S. Blumenthal-Barby

**Affiliations:** 1 Center for Medical Ethics and Health Policy, Baylor College of Medicine, One Baylor Plaza MC: 420, Houston, TX 77030 USA; 2 Division of Heart Failure, Houston Methodist Hospital, Smith Tower, 6550 Fannin St., Ste 1901, Houston, TX 77030 USA; 3 Baylor St. Luke’s Medical Center, Texas Heart Institute, 7200 Cambridge Street, Ste 6C, Houston, TX 77030 USA; 4 Duchossois Center for Advanced Medicine – Hyde Park, University of Chicago Medicine, 5758 S. Maryland Ave., Chicago, IL 60637 USA; 5 INTREGIS Advanced Cardiac Care, 3400 N.W. Expressway, Bldg C. Suite 200, Oklahoma City, OK 73112 USA; 6 Division of Cardiology, Montefiore Medical Center, Bronx, NY 10467 USA; 7 Miami Transplant Institute, University of Miami Health System, Miami, FL 33136 USA; 8 Cardiovascular Medicine, Brigham and Women’s Hospital, 75 Francis St., Boston, MA 02115 USA; 9 Aurora St. Luke’s Medical Center, 2900 W Oklahoma Ave, Milwaukee, WI 53215 USA; 10 Asheville Cardiology Associates, 5 Vanderbilt Park Dr., Asheville, NC 28803 USA; 11 Columbia Presbyterian Medical Center, Columbia University Irving Medical Center, 622 West 168th St., Room 129, New York, NY 10032 USA; 12 Department of Health Services Research, Division of Cancer Prevention and Population Services, University of Texas MD Anderson Cancer Center, 1515 Holcombe Blvd., Unit 1465, Houston, TX USA; 13 Miller Family Heart and Vascular Institute, Cleveland Clinic, 9500 Euclid Ave., Cleveland, OH 44195 USA

**Keywords:** Implementation success, Facilitators and barriers, Decision support intervention, Principal components analysis

## Abstract

**Background:**

A central goal among researchers and policy makers seeking to implement clinical interventions is to identify key facilitators and barriers that contribute to implementation success. Despite calls from a number of scholars, empirical insights into the complex structural and cultural predictors of why decision aids (DAs) become routinely embedded in health care settings remains limited and highly variable across implementation contexts.

**Methods:**

We examined associations between “reach”, a widely used indicator (from the RE-AIM model) of implementation success, and multi-level site characteristics of nine LVAD clinics engaged over 18 months in implementation and dissemination of a decision aid for left ventricular assist device (LVAD) treatment. Based on data collected from nurse coordinators, we explored factors at the level of the organization (e.g. patient volume), patient population (e.g. health literacy; average sickness level), clinician characteristics (e.g. attitudes towards decision aid; readiness for change) and process (how the aid was administered). We generated descriptive statistics for each site and calculated zero-order correlations (Pearson’s *r*) between all multi-level site variables including cumulative reach at 12 months and 18 months for all sites. We used principal components analysis (PCA) to examine any latent factors governing relationships between and among all site characteristics, including reach.

**Results:**

We observed strongest inclines in reach of our decision aid across the first year, with uptake fluctuating over the second year. Average reach across sites was 63% (s.d. = 19.56) at 12 months and 66% (s.d. = 19.39) at 18 months. Our PCA revealed that site characteristics positively associated with reach on two distinct dimensions, including a first dimension reflecting greater *organizational infrastructure and standardization* (characteristic of larger, more established clinics) and a second dimension reflecting positive *attitudinal orientations,* specifically, openness and capacity to give and receive decision support among coordinators and patients.

**Conclusions:**

Successful implementation plans should incorporate specific efforts to promote supportive and mutually informative interactions between clinical staff members and to institute systematic and standardized protocols to enhance the availability, convenience and salience of intervention tool in routine practice. Further research is needed to understand whether “core predictors” of success vary across different intervention types.

## Background

A central goal among researchers and policy makers seeking to implement clinical interventions is to identify facilitators and barriers that contribute to implementation success. These factors often operate across multiple levels, including at the site-level (e.g. patient volume) [1], staff-level (e.g. turnover, motivation and buy-in) [2] and patient-level (e.g. patient health and literacy, language barriers) [2, 3]. Understanding how these diverse factors interact to impact on implementation outcomes is one of the primary challenges to designing effective implementation strategies, and the *raison d’etre* for theoretical frameworks like the Behavior Change Wheel, the Theoretical Domains Framework, and similar models that seek to characterize interventions and link them to specific target behaviors [4–6]. However, while conceptual models such as these can be useful for implementation planning, what researchers continue to lack are real-world, empirical examinations of site “profiles” that map onto implementation outcomes of varying success. What set of features facilitate a clinical intervention, such as an evidence-based decision aid, to be successfully implemented and sustained at some sites and not others?

Which factors are associated with successful implementation may indeed vary according to the nature of an intervention. However, few empirical data yet exist to evaluate this claim, or to systematically examine to what degree site characteristics vary while still contributing to implementation successes. Likewise, little is known about whether certain site characteristics predicting success regularly overlap to form a constellation of “core implementation success predictors” applicable across interventions. These gaps constitute a continuing impediment to understanding factors contributing to implementation success and its broader impacts on shared decision making (SDM) [7].

The importance and enduring challenge of identifying factors contributing to the successful “reach” of an intervention must be understood within a larger context of the “implementation gap” described by Gayer et al. [8] in the uptake of evidence-based decision aids compared to their use in real-world settings. Decision aids (DAs) are tools designed to increase patient knowledge about risks and benefits of treatment alternatives, help patients clarify what is important to them in making a decision, and to increase patient engagement in shared decision making. The utility of DAs rests on over two decades of efficacy research [9], yet their uptake in practice continues to be limited [10]. A systematic review by Elwyn et al. (2013) attributed this implementation gap to indifference on the part of health care professionals, stemming from a lack of confidence in the content of decision support interventions and concern about disruption to established workflows [7]. More recently, Scholl et al. (2018) recommended more closely examining how organizational-level factors (leaders, culture, resources, priorities, team dynamics and workflows) and system-level characteristics (policies, clinical guidelines, incentives, culture, education and licensing) and their interactions influence implementation success [1]. Despite calls from a number of scholars, empirical insights into the complex structural and cultural predictors of why DAs become routinely embedded in health care settings remains limited and highly variable across implementation contexts [7, 11–15]. Further, few studies explicitly compare feature profiles of different implementation sites with varying levels of success in order to gain insight into what site attributes, practices or attitudinal orientations facilitate implementation success [16, 17]. In this paper we offer results from a 9-site project to disseminate and implement a validated DA for patients considering left ventricular assist device (LVAD) therapy for advanced heart failure [18–20].

### Description of implementation project

We tested our LVAD DA in a multisite randomized control trial (RCT) between 2015 and 2017 and found it significantly increased LVAD knowledge [21]. Once validated, we sought to implement the DA into clinical practice. The goals of our implementation project were to (1) Build capacity with key clinicians (physicians and LVAD nurse coordinators) to implement our LVAD DA through an initial “Capacity Building Webinar” and reinforcement sessions [22]; (2) Collaborate with “physician champions” to support LVAD nurse coordinators in their efforts to implement the DA during patient education and to use the DA themselves; and (3) Provide ongoing support to LVAD coordinators to facilitate development of sustainable practices for long-term DA use in their programs. The implementation setting includes nine U.S. hospitals, including five that participated in our original RCT of the DA and four that had no prior experience with the LVAD DA [20]. None of the nine sites were actively using the DA at the beginning of the dissemination and implementation (D&I) project due to staff turnover since the RCT. We engaged LVAD nurse coordinators as primary staff to disseminate and review the DA with patients. Coordinators generally provide LVAD education and have frequent contact with patient candidates during LVAD evaluation.

Evaluation of dissemination and implementation (D&I) progress and success is based on the RE-AIM framework (reach, effectiveness, adoption, implementation, maintenance) [23], tracking outcomes using a 10-item “Implementation Tracking Sheet” (ITS) completed for each DA use which measures fidelity to the intended use of our DA in the context of SDM. Among the variables in the RE-AIM framework, reach (percentage of eligible patients receiving the intervention) is our primary indicator and a widely recognized outcome measure for gauging the success of implementation.

In an attempt to better understand which site features contribute to greater reach we conducted brief surveys with LVAD physicians and coordinators to identify objective site characteristics (e.g. patient volume, patient sickness level) and subjective orientations (e.g. clinician readiness for change; coordinator/clinician satisfaction with the DA; perceived integration with existing educational materials) with the potential to impact implementation success).

## Methods

### Participants and variable selection

We conducted a brief online survey with LVAD coordinators (December 2019–March 2020) across 9 clinical sites currently participating in our 2-year implementation project, selected on the basis of our previous relationships with these clinics while developing and testing our DA. The purpose of the survey was to collect information from healthcare professionals (reporting to the best of their knowledge) at participating sites about characteristics of their clinical sites and patient populations, as well as their attitudes towards and use of the DA. The survey was administered using Microsoft Forms once per respondent over months 14–17 in order to capture site dynamics representative of and relevant to both of the cross-sectional time points (months 12 and 18) at which reach was assessed. Participants were notified in advance that they would be sent a $75 gift card upon completion of the survey. While individual respondents remained anonymous, their degree of anonymity was limited by their identification of their clinical site, which was needed to match their responses to reach performance.

Variables explored in the survey are reported in Table 1 and include site characteristics at the level of the *organization* (volume of annual patient LVAD evaluations and LVAD implants); *patient* (perceived average patient sickness level; perceived average health literacy and language barriers); *clinician* (experience; workload; degree of coordinator interaction with physicians; attitudes towards and use of DA versus standard education; readiness for change; distribution of DA administration responsibilities across clinical staff); and *process* (e.g. standardization of DA administration; integration of DA with existing educational materials).

**Table 1:** Clinical site variables explored

Variable type	Variable construct	Question item
Organizational	Pt Volume: Implants	In the last year approximately how many LVAD implants were placed at your hospital?
	Pt Volume: Evaluations	In the last year, approximately how much new evaluations for LVAD did your hospital have?
Patient	Pt Sickness Level	Compared to other hospitals, do you think the patients your hospital evaluates are more or less sick?
	Pt Health Literacy	Compared to other hospitals, do you think the health literacy of patients evaluated for LVAD is greater or lesser?
	Pt Language Barriers	How frequently are you unable to use the LVAD decision aid due to language barriers?
Clinician/Staff	Experience as LVAD Pt Educator	For how many years have you worked as an LVAD coordinator/educator/engineer?
	Time Spent on LVAD Pt Education	What portion of your time is spent on LVAD Education?
	Use of DA Compared to Other Clinicians	Compared to other staff on your team, do you use the decision aid more, less, or about the same?
	Coordinator-Clinician Interaction	How frequently do you talk with the physicians at your hospital regarding a patient’s LVAD evaluation?
	Satisfaction with DA	How satisfied are you with the LVAD decision aid as a resource for patient education?
	Satisfaction with Standard Education	Before you started using the LVAD decision aid, how satisfied were you with the LVAD education materials provided by your hospital?
	Readiness for Change (ORIC)	Respondent’s score on the Organizational Readiness for Instituting Change scale. Organizational readiness is broadly defined by members’ psychological and behavioral preparedness to implement organization change
Process	Standardization of DA Use	Do you give patients the LVAD decision aid at the same point during education and evaluation for candidacy?
	Integration of DA with Standard Education	How do you use the LVAD decision aid with your hospital’s existing (before decision aid) education materials?
Outcome	Reach: 12 months	Proportion of eligible individuals who received the DA by 12 months
	Reach: 18 months	Proportion of eligible individuals who received the DA by 18 months

We used “reach” from the widely used RE-AIM model as our primary outcome measure of implementation [23], defined as the proportion of eligible patients (in our case, patients under clinical evaluation for LVAD therapy) who received a DA before completing clinical evaluation and/or making a treatment choice. The decision to include the range of variables outlined above (e.g. organizational level, patient level, etc.) is based on a literature review of potential factors influencing reach [1, 17, 24–26]. For each clinical site, reach was calculated at 12 and 18 months into the implementation process. Each site submitted an ITS for each patient receiving the LVAD DA which is how we tracked the number of patients receiving a DA. Sites submitted the ITS immediately after using the DA with a patient or in aggregate for all monthly patients at the end of the month. Each month sites also reported their total number of eligible patients (patients under LVAD evaluation). The number of ITSs received was divided by the total number of evaluations to determine reach.

Because we did not want to impose an arbitrary threshold by which we considered a clinical site to have achieved implementation “success,” we sought instead to understand reach scores in a relative rather than absolute way and thus categorized each site in relation to the mean across sites at each time point (see “Results” section). Specifically, reach was categorized as *high* (≥ one standard deviation above the reach mean at each time point), *medium* (between one standard deviation above and below the reach mean) or *low* (below one standard deviation below the mean). For example, if mean reach at 12 months was 63% and the standard deviation was 19%, “low” reach would be characterized as any reach score at or below 44% (i.e. less than one standard deviation below the mean, or 63–19 = 44%). We also categorized reach this way to better correspond to the structure of our survey responses (e.g. high, medium/neutral, low).

We measured respondents’ readiness to implement change, broadly defined by individuals’ psychological and behavioral preparedness to implement organizational change, using the widely endorsed Organizational Readiness for Implementing Change (ORIC) scale [27]. This scale examines the degree to which individuals are likely to initiate change, exert greater effort, and exhibit greater persistence in implementing an intervention. Responses were scored according to the original authors’ instructions, with positive scores indicating greater readiness, and sites characterized as above (*high*) or below (*low*) or between (*medium*) one standard deviation from the mean of 51.5 (out of 60). These and all other variables are valenced with high scores indicating greater/more of the variable unit.

### Analysis

Surveys and reach data were recorded and analyzed in Excel (XLStat 2020.3.1.4). We generated descriptive statistics for each site, with survey scores from multiple clinicians from a single clinic collapsed by averaging and rounding to the nearest integer to form one score per variable per clinic. We calculated zero-order correlations (Pearson’s *r*) between all multi-level site variables including cumulative reach at 12 months and 18 months for all sites, and principal components analysis (PCA) to examine any latent factors governing relationships between and among all site characteristics, including reach.

## Results

### Implementation success outcomes (reach)

**Fig. 1:**
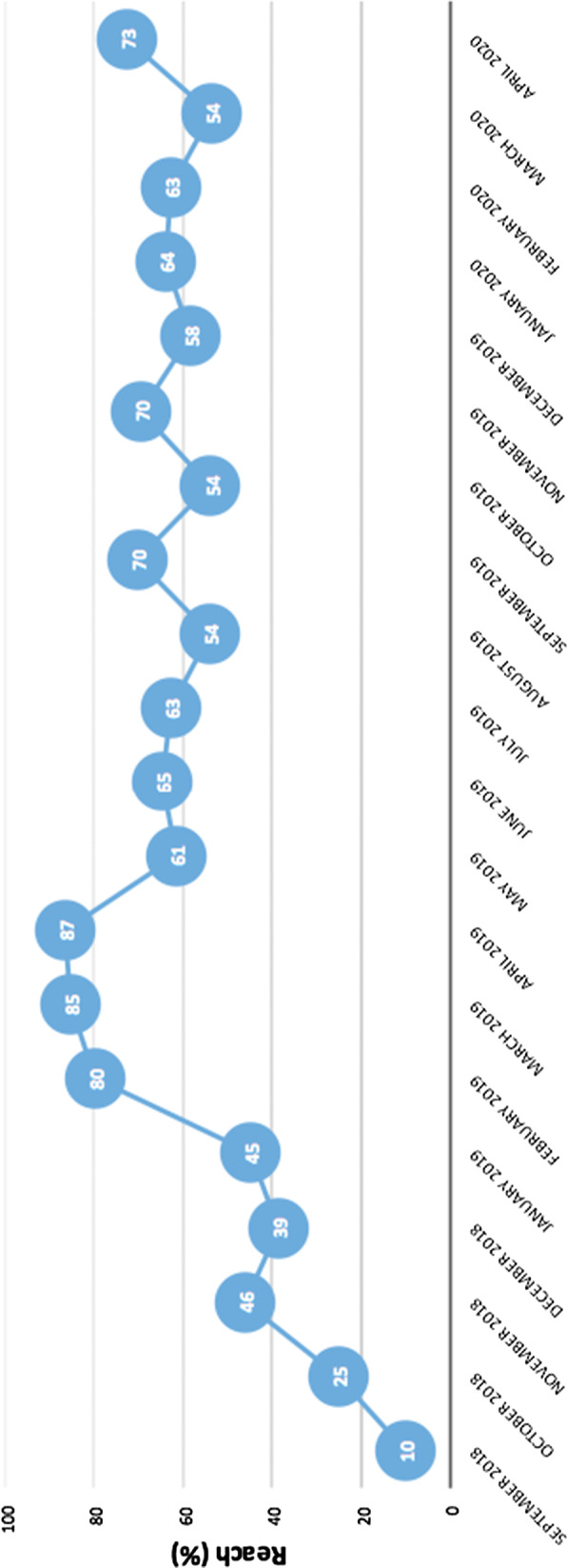
Changes in average reach levels across clinical sites over a period of 18 months (November 2019–April 2020) following initial implementation (two months consisting of orientation and startup) in September–October 2018

We observed strongest inclines in reach of our decision aid across the first year, with the frequency of uptake fluctuating over the second year. Figure 1 presents changes in average reach levels across clinical sites over a period of 18 months (November 2019-April 2020) following initial implementation (two months consisting of orientation and startup) in September–October 2018. Average reach across sites was 63% (s.d. = 19.56) at 12 months and 66% (s.d. = 19.39) at 18 months. Three out of nine sites (33%) reported high reach at both time points. Peaks in average reach were highest in months 6–8 (February-April 2019) and at the end of 18 months (April 2020). Lower reach levels were observed over the summer months as well as in October and December of 2019 (leading up to holidays) and in March 2020 (coinciding with rising cases of COVID-19 in the United States).

### Organizational characteristics of clinical sites and healthcare professionals

Descriptive site characteristics for all nine sites are presented in Table 2. Average reach scores at both 12- and 18-month time points only included eight sites, as one site did not achieve sufficient implementation startup (i.e. the site was unable to consistently provide reach data). Most sites (44% and 56%, respectively) reported a moderate patient volume of 31–50 implants per year and a high (n =  > 100) volume of evaluations. Most (78%) respondents characterized their patient populations as “more sick” compared to those at other LVAD programs, and most (78% and 56%, respectively) said their patients had about the same literacy and English language proficiency as other sites. Over half of respondents (56%) reported 0–4 years of experience with LVAD patient education and 67% said that their work duties are devoted only in part (“some of my time” versus “all” or “little”) to patient education. Other duties included administrative responsibilities and clinical care for other types of heart failure patients. Over half (56%) reported using the DA about the “same amount” as other clinical staff involved with LVAD education.

**Table 2:** Descriptive statistics for participating clinical sites (n = 9)

Variable type	Characteristic	Percentage
*Organizational Variables*		
Patient Volume: Implants		
	11–30 implants	33%
	31–50 implants	**44%**
	> 50 implants	22%
Patient Volume: Evaluations		
	36–50 evaluations	33%
	51–100 evaluations	11%
	> 100 evaluations	**56%**
*Patient Variables*		
Patient sickness level		
	Less sick	0%
	About the same	22%
	More sick	**78%**
Patient health literacy		
	Lower health literacy	22%
	About the same health literacy	**78%**
	Higher health literacy	0%
Patient language barriers		
	Rarely	44%
	Sometimes	**56%**
	Often	0%
*Clinician/Staff Variables*		
Years of experience as VAD coordinator		
	0–4 years	**56%**
	5–9 years	33%
	≥ 10 years	11%
Average time spent on LVAD patient education		
	Little of my time	11%
	Some of my time	**67%**
	All of my time	22%
Use of decision aid compared to other clinical staff		
	Less than others	11%
	Same amount as others	**56%**
	More than others or only user	33%
Level of LVAD coordinator and physician interaction		
	Rarely	0%
	Sometimes	11%
	Frequently or always	**89%**
Satisfaction with decision aid		
	Somewhat or very dissatisfied	0%
	Neutral	22%
	Somewhat or very satisfied	**78%**
Satisfaction with standard education materials		
	Somewhat or very dissatisfied	22%
	Neutral	22%
	Somewhat or very satisfied	**56%**
Readiness for Change (ORIC)		
	Less ready	22%
	Moderately ready	33%
	Very ready	**44%**
*Process*		
Standardization of decision aid (DA) use		
	Rarely	0%
	Sometimes	11%
	Frequently	**89%**
Integration of decision aid (DA) with standard education (SE) materials		
	Use SE more than the DA	22%
	Use DA and SE about same	**44%**
	Use DA exclusively or more than SE	33%
*Outcome*		
Reach: 12 months*^		
	Sites with **low** reach (≤ *x̅)*	22%
	Sites with **medium** reach 44%-82% (between 1 s.d above/below *x̅*)	44%
	Sites with **high** reach ≥ *x̅*	**33%**
Reach: 18 months**^		
	Sites with **low** reach (≤ *x̅)*	22%
	Sites with **medium** reach 46%-84% (between 1 s.d above/below *x̅*)	44%
	Sites with **high** reach ≥ *x̅*	**33%**

* x̅ = 63%; s.d. = 19%

**x = 65%; s.d. = 19%

^ Does not include reach from one site who did not achieve start-up

May not sum to 100% due to rounding

Highest percentage values are bolded for each variable

### Clinician attitudes and readiness for implementation

Over three quarters (78%) of respondents reported feeling somewhat or very satisfied with the DA, while only 56% said the same about standard education materials. Just under half (44%) demonstrated high readiness for change based on responses to the ORIC scale, with 22% reporting low readiness.

### Process-related barriers/facilitators

A majority (89%) of sites reported a high level of standardization in DA use. Almost half (44%) of sites reported moderate integration of the DA with existing standard education materials (i.e. using them about the same amount) as opposed to using the DA more (33%) or less (22%) than standard education materials.

### Associations between site characteristics and reach

Table 3 presents zero-order correlations between site characteristic variables, with significant correlations (p ≤ 0.05) bolded. We observed a significant negative correlation between clinician experience and the proportion of their duties or time spent on patient LVAD education (r = −0.74; p ≤ 0.05). We also found significant positive correlations between the level of coordinator-physician interaction and a clinician’s use of the DA compared to other staff (r = 0.71; p ≤ 0.05); between the perceived health literacy of a patient population and coordinator/clinician satisfaction with standard education materials and procedures (r = 0.88; p ≤ 0.05); standardization of DA use within a clinical site and a clinician’s more frequent use of the DA compared to other clinicians (r = 0.88; p ≤ 0.0571); and between a site’s number of LVAD patient evaluations and implants (r = 0.86; p ≤ 0.05). We also found a perfect correlation between *overall* average reach at 12 months and at 18 months (r = 1.0, p ≤ 0.05), though individual sites varied in the consistency of their reach over these two time points.

**Table 3:**
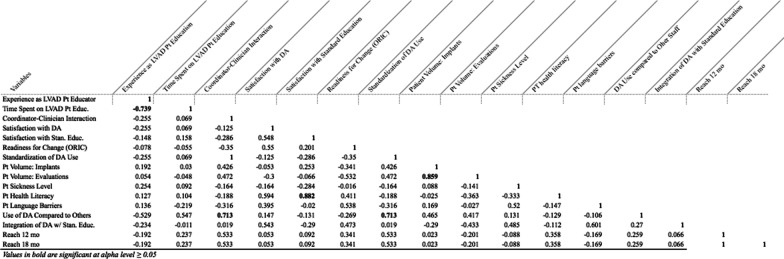
Site characteristic (Pearson’s r) correlations across 9 LVAD clinics

A principal components analysis revealed a cluster of site characteristics positively associated with reach on two distinct dimensions, which we interpreted to reflect a structural/organizational dimension (F2) and an attitudinal dimension (F1). Reach was particularly central on factor two (F2), where we observed high positive associations (i.e. factor loadings ≥ 0.66) with reach for variables including perceived patient health literacy, clinician/coordinator satisfaction with the DA, and readiness for change (see Table 7). Another dimension (F1) accounted for roughly equivalent variance (see Table 4) and showed high positive associations (factor loading ≥ 0.50) for variables including level of coordinator-physician interaction, standardization of DA use, use of DA compared to other staff, and high volume of patient evaluations and implants (see Table 5). These variables appeared together with reach at 12 and 18 months (factor loadings = 0.42) (Table 6).

**Table 4:** Principal component analysis: eigenvalues for factors 1–5

	F1	F2	F3	F4	F5
Eigenvalue	4.30	3.45	2.59	1.98	1.73
Variability (%)	26.9	21.6	16.2	12.4	10.8
Cumulative %	26.9	48.4	64.6	77.0	87.8

**Fig. 2:**
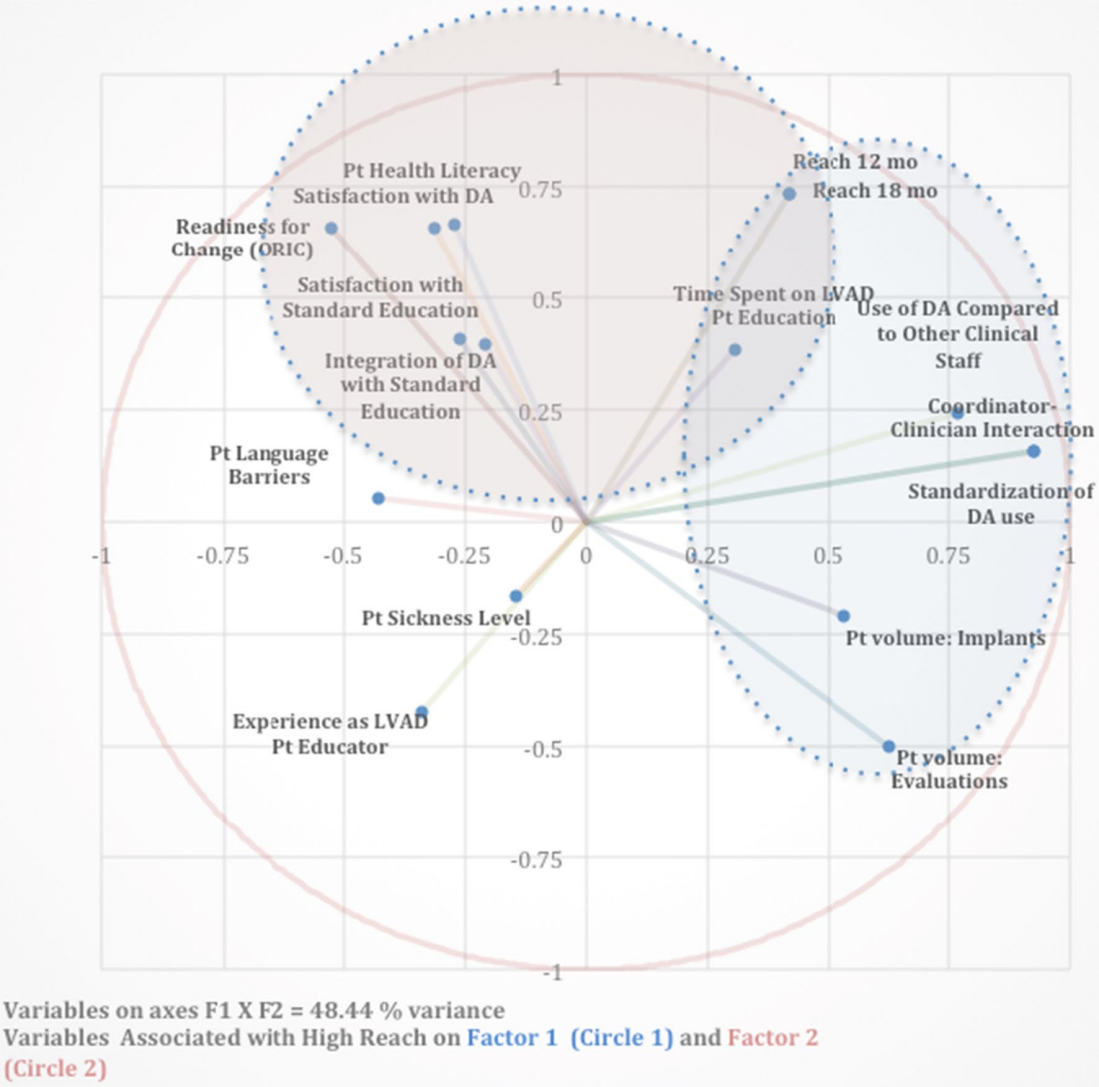
Multidimensional scale of site characteristics in relation to reach

The Kaiser–Meyer–Olkin co-efficient, which measures the proportion of variance among variables that might be due to common variance, was moderately low (0.38), indicating that, overall, site variables are only loosely related (are independent of one another), or that more sites must be sampled in order to better understand how site variables are associated. Factors 1 and 2 pictured in the multidimensional scale in Fig. 2 account for 48% of the variance, leaving over 50% of the variance unexplained by the measured variables.

## Discussion

Strongest inclines in uptake and reach of our decision aid occurred across the first year, with frequency of uptake fluctuating over the second year. That the largest of these fluctuations coincided with typical seasonal/holiday shifts (October-December 2019) as well as the sharp rise of the COVID-19 pandemic in the United Stated (March 2020), suggests that larger situational factors at the national or even global level can influence reach. At a more local level, however, we discovered key site characteristics that appear to have important associations with reach. These site characteristics form two distinct constellations of variables that are non-overlapping, apart from their pivotal associations with reach, suggesting there may be more than one effective pathway to implementation success.

On one dimension, we found that reach is associated with site characteristics indicating greater *organizational infrastructure and clinical standardization*, variables typical of larger, more established clinics. Specifically, this constellation of traits includes greater volume of patient evaluations and implants, more frequent physician involvement with LVAD nurse coordinators, more highly specified role distribution (i.e. designated personnel for delivering patient education and decisional support), and greater standardization of patient education protocols.

Meanwhile, on a separate, second dimension, reach showed strong associations with a group of variables suggesting the importance of *attitudinal orientation,* including openness and capacity to give and receive decision support among coordinators and LVAD candidates, respectively. On this factor, high reach was associated with greater patient health literacy, greater coordinator/clinician satisfaction with the DA, and greater readiness for change. Thus, a patient’s ability to understand the health information conveyed during patient education, coupled with a coordinator’s more positive attitudes toward the DA and a broader sentiment among other staff of readiness for improvements, may together facilitate greater uptake of decision support.

### The attitudinal dimension: pro-change attitudes and motivation

An openness to provide and receive decision support has long been recognized as a crucial ingredient in the uptake of shared decision making tools [1, 15, 28]. Indeed, motivation of health professionals was found to be a top factor in a systematic review of barriers and facilitators of implementing SDM, including DAs, in practice [13]. However, generating “buy-in” to the theoretical and practical importance of providing decision support is one of the most formidable challenges to effective implementation. A number of documented implementation approaches center on providing education to clinicians and staff members [28–32]. However, the assumption that with knowledge comes motivation may be erroneous, and some researchers [33–35] have pointed out that education- or persuasion-based approaches may be ineffective if they do not also address individuals’ diverse motivations for engaging with an intervention. Such approaches are likely to encounter resistance from clinical staff’s default, habitual ways of thinking and behaving (sometimes called ‘‘bounded rationality’’) [36].

To address the need for fostering incentives that are internalized as positive, reflexive attitudes towards an intervention, we have outlined elsewhere a series of tools designed to positively affect clinicians’ and staff members’ motivations for using a DA [37]. This toolkit, called MINDSPACE, draws from robust insights in behavioral economics to offer empirically supported strategies for implementers seeking to initiate small attitudinal, emotional, or behavioral responses that can collectively help bring about lasting, positive behavioral change. One optimistic insight is that attitudes and orientations towards shared decision-making and positive change may be more easily altered than structural variables, such as patient volume or available time spent on patient education, and are likely to be less economically and logistically costly to modify [37]. For this reason, implementation scientists seeking to foster clinical characteristics associated with successful implementation of decision support tools may look towards strategies to generate staff interest in and motivation to use SDM tools. This recommendation is widely cited by an already sizable implementation science literature and empirically supported by our findings [1, 15, 28].

### The structural dimension: organizational size, standardization and interaction

A second implementation approach suggested by our findings is to consider how larger clinics may serve as models for smaller clinics, specifically with regard to organizational characteristics that may be *normatively linked* to but not *necessarily dependent* on clinic size and infrastructure. For example, a close examination of our results on the first dimension reveals that the two variables most highly associated with reach include *interaction between clinicians and staff* and *level of standardization* involved in administration of our DA. Our findings show that larger clinics (with greater patient volume and number of evaluations) have greater communication and standardization of clinic procedures. This may be because, just as standardization in industry allows for economies of scale and enables markets to optimize their transactional efficiency, it is also likely that greater standardization and greater organizational guidance and oversight from physicians are features that help larger clinics to efficiently and effectively (safely) deliver services to large patient populations, particularly where staff members may be busiest and most time-constrained [38]. While these structural and organizational features may be more prevalent among larger, highly resourced clinics, they are not dependent on clinic size and may serve as goalposts for smaller clinics hoping to effectively position themselves for successful implementation.

To better understand the significance of these organizational variables and their “active ingredients” for implementation success, we turn to a growing literature in implementation science and clinical decision support. Specifically, what may account for the positive impacts of frequent interaction between clinicians and staff, and what might these interactions entail? Studies from over two decades of implementation research suggest that implementation success is typically bolstered by having at least one clinician champion to promote the use of a decision support intervention [24, 33, 39–43]. Interactions that *demonstrate support and endorsement* of a decision support tool from a respected clinician (especially a physician) “messenger” can offer the extra incentive needed for a clinical staff member to prioritize and recognize the value of an intervention [37]. The critical role of clinician champions has also recently been highlighted by Berry et al. [42], who found that designating a clinical lead for implementation helped to address staff misunderstandings about which contexts and resources were best suited for administering decisional support. Brinkman et al. [43] likewise found that implementation was facilitated by buy-in from physicians about the value of SDM and formal training workshops for clinical staff implementing decision support. Further, a study by Uy et al. [16] similarly identified physician support as “crucial” to the distribution success of patient decision supports. In our own experience, we observed that communication between and among clinicians and coordinators offered important opportunities to communicate buy-in from clinicians to coordinators and other members of the clinical team who prioritized use of our DA as a result.

In our implementation project, we observed that physician buy-in was most crucial at the first stages of implementation (orientation and startup) to demonstrate support of our DA from clinical leaders and to provide the leverage necessary to make workflow changes or transition towards integration with or (in rarer cases) replacement of existing patient education materials. After this initial startup period, clinicians were called on less often to actively demonstrate their support as coordinators increasingly sustained their own momentum and expertise in using the DA. Thus, we recommend enlisting clinician support at early stages of implementation to leverage their practical knowledge and influence.

The studies cited above indicate that frequent clinician and clinical staff interaction may positively impact on implementation success by providing incentives and motivations for clinical staff engagement. However, because we did not see this variable appear alongside the attitudinal and motivational dimension revealed by our PCA, we must consider that other aspects of interaction may be equally important. In particular, interaction between clinical team members may also help to *communicate practical support and guidelines* for how to undergo implementation in ways that are consistent with site-specific goals and available resources. Tietbohl et al. [44] showed that clinical sites with high implementation success exhibited frequent, timely and accurate communication between clinicians and clinical staff, while lower performing clinics had more contentious relationships and inadequate communication. The nature of these interactions involved conveying practical guidance, troubleshooting and ongoing feedback to keep staff apprised of their distribution progress. Interaction may thus be associated with greater standardization because *interaction provides a forum for communicating practical, concrete steps* towards standardizing use of an intervention in practice. Cuypers et al. [45] similarly found that success in implementing a decision support tool was dependent on integrating clinical team members not only to influence their motivation but also to help navigate the clinical infrastructure needed to integrate and standardize use of an intervention into daily work patterns.

The importance of making an intervention “visible” in routine practice can also enhance use of decision support and systematize implementation by providing reminders to use the tool, encouraging strategic placement of visual cues in the workspace such as distribution checklists on computers, providing pre-written scripts with talking points for staff administering the DA, and scheduling timely feedback sessions for staff to discuss implementation progress [17]. In a further example from our own project, one of our highest-reach sites used our DA as part of a more extensive clinical evaluation and patient education checklist instituted and championed by the director of the LVAD program. Other examples from the literature (e.g. Scalia et al. [33]) suggest that standardizing use of a decision support tool as part of a mandate or milestone completion expected by a clinical supervisor can result in improved interactions between patients and healthcare professionals, with patients asking more questions and feeling more satisfied and empowered in decision making. Evidence suggests that interaction with clinician champions can also provide structural insights about how best to systematize procedures within existing flow to facilitate referral, ordering and administration of DAs to patients [10, 24, 33, 42].

Examples of how to effectively standardize or systematize implementation to promote use of a DA include systematically identifying eligible patients to receive decision support tools in advance of their clinical visits [33], offering referral or ordering options in a patient’s electronic health record to ensure availability of a DA for patients to review ahead of their clinic visits [43], and having a DA readily accessible at a standardized place and time of decision making [24]. Based on our results and experience, contextualized by these previous studies, we thus offer an additional concrete recommendation to standardize the time, place and process for administering decision support using checklists or “kits” to ensure their availability, salience and convenience for routine use, particularly in fast-paced, busy clinical settings.

### The importance of multi-directional communication

We also observed that physicians are not always the only individuals to conceive of or initiate pro-implementation changes, and that the direction of effective communication is not always “top-down” (i.e. physician to coordinator) but multidirectional. Physicians can also learn from coordinators, nurses and other clinical staff who work in more regular proximity with patients about other “ground-level” considerations that are important for administering decision supports effectively or meaningfully in the daily clinic setting. In our own project, we observed that certain LVAD coordinators were the de facto champions of using our DA in practice and were effective at generating awareness and support from clinicians and other clinical staff. Coordinators harbor a wealth of observational experience that inform practical suggestions and solutions for implementation success at the patient level. Thus, while physician champions may be more suited to authorize and gain higher-level buy-in for infrastructural changes, bi-directional communication with nurse coordinators and other clinical staff can help to generate “grass roots” support and practical insights for implementing an intervention effectively in routine practice. We believe that our findings offer further support for the importance of fostering effective communication channels between physicians and staff in order to integrate different and equally important perspectives on engaging stakeholders at multiple levels (administrative, clinician-, staff- and patient-level). Based on these insights, we thus offer a final recommendation: to create forums for frequent exchange of perspectives and practical information across multiple roles, prioritizing “on the ground” insights (e.g. from coordinators) within a larger context of organizational resources and constraints (conveyed by clinicians or administrative personnel). The recommendations discussed above are listed in Table 7 as key takeaways for researchers interested in empirical insights to inform implementation science theory, and/or for clinicians and clinical programs seeking to better position themselves for implementation success.

**Table 5:** Variable factor loadings (F1–F5) and % contributions ordered by F1 loadings

	F1	%	F2	%	F3	%	F4	%	F5	%
Coordinator-Clinician Interaction	**0.93**	**19.9**	0.16	0.70	0.09	0.31	0.02	0.02	− 0.23	3.02
Standardization of DA Use	**0.93**	**19.9**	0.16	0.70	0.09	0.31	0.02	0.02	− 0.23	3.02
Use of DA Compared to Other Clinical Staff	**0.77**	**13.7**	0.24	1.67	0.32	4.00	0.27	3.72	0.32	6.09
Pt volume: Evaluations	**0.63**	**9.09**	− 0.50	7.28	− 0.15	0.90	0.50	12.45	0.05	0.13
Pt volume: Implants	**0.53**	**6.59**	− 0.21	1.26	− 0.17	1.16	0.77	29.81	− 0.08	0.36
Reach 18 mo	**0.42**	**4.07**	0.73	15.50	− 0.06	0.15	− 0.19	1.84	− 0.43	10.7
Reach 12 mo	**0.42**	**4.07**	0.73	15.50	− 0.06	0.15	− 0.19	1.84	− 0.43	10.7
Time Spent on LVAD Pt Education	**0.30**	**2.15**	0.38	4.27	0.07	0.20	− 0.11	0.59	0.71	28.7
Pt Sickness Level	− 0.14	0.48	− 0.16	0.78	0.66	16.9	0.20	2.00	− 0.15	1.29
Integration of DA with Standard Education	− 0.21	1.02	0.39	4.48	0.83	26.4	0.14	1.02	− 0.04	0.07
Satisfaction with Standard Education	− 0.26	1.60	0.41	4.83	− 0.68	17.7	0.48	11.7	0.17	1.69
Pt Health Literacy	− 0.27	1.73	0.67	12.81	− 0.61	14.4	0.22	2.45	− 0.01	0.01
Satisfaction with DA	− 0.31	2.30	0.66	12.49	0.06	0.13	0.48	11.7	0.23	3.18
Experience as LVAD Pt Educator	− 0.34	2.70	− 0.42	5.18	− 0.28	3.04	0.25	3.05	− 0.68	26.4
Pt Language Barriers	− 0.43	4.25	0.05	0.07	0.58	12.9	0.59	17.5	− 0.18	1.80
Readiness for Change (ORIC)	− 0.53	6.45	0.66	12.45	0.19	0.08	0.09	0.30	0.30	2.68

Bold values represent positive factor loadings ≥ 0.30 on Factors 1 and 2, respectively

**Table 6:** Variable factor loadings (F1–F5) and % contributions ordered by F2 Loadings

	F1	%	F2	%	F3	%	F4	%	F5	%
Reach 18 mo	0.42	4.07	**0.73**	**15.5**	− 0.06	0.15	− 0.19	1.84	− 0.43	10.7
Reach 12 mo	0.42	4.07	**0.73**	**15.50**	− 0.06	0.15	− 0.19	1.84	− 0.43	10.7
Pt Health Literacy	− 0.27	1.73	**0.67**	**12.8**	− 0.61	14.4	0.22	2.45	− 0.01	0.01
Satisfaction with DA	− 0.31	2.30	**0.66**	**12.5**	0.06	0.13	0.48	11.7	0.23	3.18
Readiness for Change (ORIC)	− 0.53	6.45	**0.66**	**12.5**	0.19	1.40	0.08	0.30	− 0.22	2.68
Satisfaction with Standard Education	− 0.26	1.60	**0.41**	**4.83**	− 0.68	17.7	0.48	11.7	0.17	1.69
Integration of DA with Standard Education	− 0.21	1.02	**0.39**	**4.48**	0.83	26.4	0.14	1.02	− 0.04	0.07
Time Spent of LVAD Pt Education	0.30	2.15	**0.38**	**4.27**	0.07	0.20	− 0.11	0.59	0.71	28.7
Use of DA Compared to Other Clinical Staff	0.77	13.7	0.24	1.67	0.32	4.00	0.27	3.72	0.32	6.09
Coordinator-Clinician Interaction	0.93	19.9	0.16	0.70	0.09	0.31	0.02	0.02	− 0.23	3.02
Standardization of DA Use	0.93	19.92	0.16	0.70	0.09	0.31	0.02	0.02	− 0.23	3.02
Pt Language Barriers	0.043	4.25	0.05	0.07	0.58	12.9	0.59	17.5	− 0.18	1.80
Pt Sickness Level	− 0.14	0.48	− 0.16	0.78	0.66	16.9	0.20	2.00	− 0.15	1.29
Pt Volume: Implants	0.53	6.59	− 0.21	1.26	− 0.17	1.16	0.77	29.8	− 0.08	0.36
Experience as LVAD Pt Educator	− 0.34	2.70	− 0.42	5.18	− 0.28	3.04	0.25	3.05	− 0.68	26.4
Pt Volume: Evaluations	0.63	9.09	− 0.50	7.28	− 0.15	0.90	0.50	12.5	0.05	0.13

Bold values represent positive factor loadings ≥ 0.30 on Factors 1 and 2, respectively

**Table 7:** Summary of recommendations for decision support implementation

Generate staff interest in and motivation to use SDM tools
Enlist clinician support at early stages of implementation to leverage their practical knowledge and influence
Standardize the time, place and process for administering decision support
Create forums for frequent exchange of perspectives and practical information across multiple roles

## Limitations

A primary limitation is that our analysis leaves over 50% of the variance unexplained by the measured variables. Further, the proportion of variance among variables that might be due to common variance, was moderately low (0.38), indicating that the variables we measured may be only loosely related and require further verification, or a larger number of cases to better understand variable associations.

A second limitation is that we measured “interaction between clinicians and staff” by asking respondents to report on *frequency* of interaction. Further, we asked about “level of standardization” with reference to *timing consistency*—that is, whether coordinators’ administered the DA at the same time in the educational process across patients. A potential shortcoming of these phrasings is that other aspects of interaction and standardization beyond frequency and timing, respectively, may be equally or more important. Greater insights are needed into which features of clinician-coordinator interaction and standardization impact on implementation success.

A third limitation is that our results are based on respondents’ perceptions and may thus not accurately reflect the actual clinic characteristics and team-wide attitudes. Further research involving a more extensive range of clinics as well as rigorous measurement of site characteristics is needed to confirm our findings.

## Conclusion

A critical goal among implementation researchers and policy makers is to identify clinical site characteristics that facilitate implementation success of a clinical intervention. Our study highlights two distinct groups of site characteristics empirically associated with greater use of a decision support tool and suggests that successful implementation plans should incorporate specific efforts to promote supportive and mutually informative interactions between clinical staff members and to institute systematic and standardized protocols to enhance the availability, convenience and salience of intervention tool in routine practice. Our results provide insights that are supported by a growing implementation science literature and may be useful for clinicians and LVAD programs seeking to better position themselves for effective integration of decision support into their patient education, or to evaluate how existing site dynamics might forecast certain implementation outcomes.

## Data Availability

The datasets used and/or analyzed during the current study are available from the corresponding author on reasonable request.

## Abbreviations

DA: Decision Aid
D&I: Dissemination and Implementation
ITS: Implementation tracking sheet
LVAD: Left ventricular assist device
PCA: Principal components analysis
RCT: Randomized Controlled Trial
SDM: Shared decision-making
